# Anemia Prevalence after Iron Supplementation among Pregnant Women in Midwifes Practice of Primary Health Care Facilities in Eastern Indonesia

**DOI:** 10.1155/2019/1413906

**Published:** 2019-10-22

**Authors:** Merry M. V. Seu, Johanes C. Mose, Ramdan Panigoro, Edhyana Sahiratmadja

**Affiliations:** ^1^Program Study of Midwifery, Division of Maternal and Child Health, Department of Public Health, Faculty of Medicine, Universitas Padjadjaran, Bandung, Indonesia; ^2^RSUD Prof. Dr. W. Z. Johannes General Hospital, Kupang, Eastern Nusa Tenggara, Indonesia; ^3^Department of Obstetrics and Gynecology, Faculty of Medicine, Universitas Padjadjaran/Dr. Hasan Sadikin General Hospital, Bandung, Indonesia; ^4^Department of Biomedical Sciences, Faculty of Medicine, Universitas Padjadjaran, Bandung, Indonesia; ^5^Research Center of Medical Genetics, Faculty of Medicine, Universitas Padjadjaran, Bandung, Indonesia

## Abstract

**Background:**

Iron deficiency anemia (IDA) in pregnant women is common, and iron supplementation is given during pregnancy to reduce birth complication. This study aimed to explore the prevalence of anemia and type of anemia after iron supplementation among pregnant women in the eastern part of Indonesia.

**Methods:**

A cross-sectional study design was conducted between January and March 2019 in three Primary Health Care (PHC) facilities at Kupang, West Timor. After consent, pregnant women who had taken their iron supplementation for at least 3 months were asked for iron pills intake by using a self-designed questionnaire and by counting the pills leftover. Complete blood count examination was performed, and the type of anemia was assessed using Shine and Lal index (SLI; MCV *∗* MCV *∗* MCH/100) to determine whether anemia was due to iron deficiency or *β*-thalassemia trait (*β*-TT). In a subset of iron tablets distributed in the PHCs, Fe-concentration was measured.

**Results:**

Of 102 pregnant women included, only 25.5% had taken the pills with a pill count of >80%. Interestingly, Fe-concentration in the pills from three different PHC facilities varied between 75% and 100%. After iron supplementation, however, anemia was detected in 34.3%, and based on SLI, 14.7% was suspected because of iron deficiency and 19.6% was suspective of *β*-TT. Of note, nonanemic pregnant women (17.6%) had also low SLI, suggesting *β*-TT or other hemoglobinopathies.

**Conclusion:**

Assessment of Shine and Lal index as the first step to screen the type of anemia in pregnant women from a limited area is of potential value, especially because Indonesia is located in the thalassemia belt area. An integrative approach and counseling among pregnant women with *β*-TT and their partners will increase thalassemia awareness and optimal birth management.

## 1. Introduction

Anemia in pregnancy is a common phenomenon in low- and middle-income countries, and it is due to a reduction in the hemoglobin concentration despite an increase in the red cell mass. Anemia may cause bleeding complications in the mother throughout the pregnancy period, in labor, and after the baby is born, as well as impaired growth in the fetus [1]. The cause of anemia in pregnant women is mainly due to iron deficiency, and the prevalence in developing countries is high [2]. Iron deficiency anemia (IDA) in pregnant women has been described as a silent killer; thus, extra iron supplementation or therapy need to be given to increase Hb [3]. Low intake of micro nutrients plays an important role; therefore, pregnant women are encouraged to consume routine iron/folate supplementation or otherwise Fe-containing food [1]. The WHO recommends oral iron supplementation every day as much as 30–60 mg to be able to meet iron requirements [2], especially in the third trimester of pregnancy [4]. In Indonesia, government efforts to prevent anemia and iron supplementation has been given for free, as a national program [5]. Although this program for administering iron supplementation has been going on for a long time, the prevalence of anemia in pregnant women is still high [6], contributing to obstetric complications.

Despite iron supplement therapy, however, an important factor in the success of IDA therapy is the adherence of pregnant women to consume iron supplementation [7]. There are many factors associated with the adherence to iron supplementation among others good knowledge and motivation of the pregnant women [8]. Because of various side effects of iron intake such as nausea, excessive vomiting, and others, some pregnant women are tended to stop iron therapy [9]. Furthermore, infections such as helminthes and malaria are endemic in the developing countries, leading to IDA in pregnant women [10, 11]. Moreover, genetic factors may also play a crucial factor in the synthesis of globin of the red blood cells [12]. Our previous study in the western part of Indonesia has shown an unexpected high number of pregnant women with hemoglobinopathy disorders, and the midwives are unaware about the screening of the carrier [13]. In light to this, there is a need to consider anemia attributable to etiologies other than IDA in developing countries.

The aim of the study was to explore the anemia prevalence among pregnant women in the eastern part of Indonesia after iron supplementation. The adherence of iron tablets intake was assessed, and the anemia type was predicted using Shine and Lal Index, whether the anemia was due to iron deficiency or suspect *β*-thalassemia trait.

## 2. Materials and Methods

### 2.1. Study Design

The study was cross sectional and descriptive analytic design, conducted between January and March 2019 in three Primary Health Care (PHC) facilities in Kupang, West Timor, eastern part of Indonesia, where malaria infection was endemic.

### 2.2. Inclusion Criteria for Pregnant Women

In brief, information about anemia in the waiting room was presented to all pregnant women who visited antenatal care in PHC facilities. The information consisted of the possibility and the effect of anemia due to iron deficiency in the pregnancy and the possible state of *β*-TT. Pregnant women with singleton pregnancy in 2^nd^ and 3^rd^ trimester of pregnancy who had taken their iron tablets for at least 3 months (90 tablets) were invited to take part in the study. After consent, a self-designed questionnaire was distributed, asking about iron pills intake in the last 30 days. Furthermore, the pregnant women were asked for complete blood count (CBC) examination. Of note, CBC was not performed at a regular base of blood examination in midwife practice at PHCs, but Hb was measured using a prick test at PHC. Pregnant women with twins, triplets or more, heart disease history, preeclampsia, or history of antepartum bleeding were further excluded.

The study protocol had been reviewed, and ethical clearance was granted by the Ethical Committee of Faculty of Medicine, Universitas Padjadjaran (no. 71/UN6.KEP/EC/2019).

### 2.3. Iron Pills Intake and Fe-Concentration Analysis

After consent, a self-designed questionnaire was distributed to assess the iron pills intake, consisting of 10 questions with dichotomous answers. Pregnant women were asked about the leftover of the pills (question 8), and the percentage of the pill intake was then calculated as follows: the pill count intake was designated as <80% when pill leftover was >6 pills of 30 pills a month, and as >80% when leftover was <6 pills of 30 pills a month. The frequency of the answers was described in percentage and were then analysed whether there was an association with the anemia state (Hb < 10.5 g/dL).

Furthermore, Fe-concentration was measured in a subset of iron pills from three Primary Health Care (PHC) facilities. In brief, Fe-concentration of 10 pills from each PHC with the same batch given to pregnant women were measured using atomic absorption spectrophotometry, conducted in Faculty of Pharmacy, Universitas Padjadjaran. The mean concentration was calculated and compared with the Fe-concentration stated in the drug facts label.

### 2.4. Anemia Status and Shine and Lal Index Analyses

Venous blood samples were drawn into a 3 ml EDTA tube and stored at 2–8°C before being transported to Prof Dr. W.Z Johannes General Hospital, Kupang. The distance between three Primary Health Care facilities and the hospital was approximately 4 km, and the time between blood drawn and measurement was about 2 hours. An automated hematology analyzer for complete blood count (CBC) examination was used (Sysmex XN-550, Japan) to measure parameters, among others Hb, MCV, and MCH. The Hb value for anemia in pregnancy was set according to modified WHO definition, published by the “South Australia Clinical Guideline for Anemia in Pregnancy,” that was normal (≥10.5 g/dL) and anemia (<10.5 g/dL) for second and third trimester [14]; the grades of anemia was categorized into mild anemia (10–<10.5 g/dL) and moderate anemia (Hb 7 to <10 g/dL) [2]. Shine and Lal index (SLI; MCV *∗* MCV *∗* MCH/100) was manually calculated. Anemia with SLI ≥ 1530 was designated as IDA, and anemia with SLI < 1530 was designated as suspective for *β*-thalassemia trait (*β*-TT) or *carrier*. A slide of peripheral blood smear was examined to assess the anemia finding further. A blood smear on the slide was also performed to examine for malaria infection because this study area was malaria endemic. The demographic and clinical characteristics were recorded, including age in risk *i.e.* age <20 years and >35 years and body mass index (BMI) in pregnancy. Although BMI values were of little significance in pregnancy, especially in the third trimester, however, BMI <25 was considered low for pregnant women in the second and third trimester [15].

### 2.5. Statistical Analyses

Data were collected into a paper-based form and inputted into predesigned data sheet. The demographic and clinical characteristic of the pregnant women, as well as the answers of the questionnaire were described and presented in frequency. The distribution of age in risk (<20 years and >35 years), education level, occupation group, gravidity, trimester, and BMI was explored whether there was significant association with anemia status in pregnancy (*Chi-Square*) with *p* value <0.05 was set as significant. The odds ratio (OR) and confidence interval (95% CI) were generated using SPSS version 22.0, licensed to *Universitas Padjadjaran*.

## 3. Results

In total, there were 164 pregnant women attending three Primary Health Care facilities between January and March 2019; of whom, 102 consented to fill in the questionnaire and gave permission for complete blood count (CBC) examination, consisting of 12 and 90 pregnant women in 2^nd^ and 3^rd^ trimester of pregnancy. None of these pregnant women were positive for malaria infection. The result showed that there were 35 of 102 (34.3%) pregnant women with low Hb (<10.5 g/dL), even though after 3 months of iron supplementation.

The age, education level, occupation status, and gravidity had no significant association with anemia status in pregnant women (Table 1). Interestingly, anemia status in pregnant women had a significant association with BMI < 25 (*p* 0.002). The odds of being anemic were four times higher in pregnant women with low (<25) BMI than those with high (≥25) BMI (OR 4.38 and 95% CI 1.68–11.39).

The distribution of questions asked among pregnant women from 10 questions showed that Q6 and Q8 had an opposite frequency with the result of Q9 and Q10 (Table 2). No significant relationship between the answers of the Q6, Q8, Q9, and Q10 with the anemia state (Table 3).

When assessing the possible factors of anemia, Fe-concentration in PHC1, PHC2, and PHC3 was measured, resulting in the average of 62.8 mg (range 61–64.8 mg), 44.4 mg (range 39.2–46.9 mg), and 44.9 mg (40.9–47.3 mg), respectively (data not shown). Of note, PHC 02 and 03 had Fe-content of 74% and 74.8%, and these had the same batch number and were further pooled into one group, resulting in Fe-content of 100% and of ∼75%, for PHC1, and PHC2 and PHC3, respectively, as summarized in Table 3. However, no significant difference was found (*p* 0.247) between the answer of Fe-concentration with the anemia state.

Furthermore, analysis of anemic pregnant women (*n*35) using SLI had shown that an SLI ≥ 1530 predicted pregnant women with iron deficiency in 14.7% (*n*15), whereas an SLI < 1530 predicted pregnant women with suspected *β*-TT or carrier in 19.6% (*n*20). Interestingly, there were other nonanemic women with low SLI (17.6%; *n*18), indicating suspected *β*-TT or other hemoglobinopathies. Thus, in total, there were 37.6% (*n*38) pregnant women with suspected *β*-TT or other hemoglobinopathies, and these women were further suggested to be checked for HbA2 analysis in a referral laboratory in other cities for confirmation because this area was of limited resource area (Table 3).

The distribution of anemia type due to IDA of suspected *β*-TT among pregnant women in second and third trimester was presented (Table 4). Because of the low number of pregnant women in second trimester, no statistical analysis was performed to compare data between second and third trimester, however, stratifying the pregnancy trimester and BMI with the type of anemia revealed that BMI < 25 tended to be associated with iron deficiency anemia, as shown Table 4, especially in third trimester. Moreover, mild anemia was tended to be more prevalent in third trimester than second trimester.

## 4. Discussion

This is the first study conducted in the midwife practice of Primary Health Care facilities in the eastern part of Indonesia exploring the type of anemia in pregnant women whether anemia is due to iron deficiency or *β*-thalassemia trait or carrier. The result shows that anemia prevalence among pregnant women in Kupang after iron supplementation is 34.3%; of whom, 14.7% is predicted based on an SLI > 1530 due to iron deficiency which is much lower than the national data [6]. The other anemia state is predicted based on an SLI < 1530 (19.6%), and this group is suspected to be *β*-TT or carrier. Together with another nonanemic pregnant with low SLI (17.6%), our study result in a total of 37.2% suspected *β*-TT or other hemoglobinopathies, which is much higher than *β*-TT data in the western part of Indonesia [12]. This interesting result needs further confirmation.

There are several risk factors that may contribute to anemia among pregnant women, such as medication adherence [16]. The knowledge of the need of iron supplementation, education, side effects such as nausea, and forgetfulness to take the pill play an important role in medication adherence among pregnant women. There are many ways to assess medication adherence using both subjective and objective measurements as reviewed by Nguyen et al. [17]. In our study, objective measures are performed, including measurement of Hb indicating anemia state of pregnant women and Fe-concentration of the pills is also measured. Interestingly, the anemia state may be confounded by other factors rather than iron deficiency anemia solely such as in the case of hemoglobinopathy. The questionnaire as subjective measurement of adherence in our study has contradictory results in Q6 and Q8 are in different percentage compared with Q9 and Q10, showing that pregnant women may give answer that in line with their expected response [17]. Although almost ∼75% of the pregnant women admit that they forget to take the pills and the pill intake of <80%, both factors have no association with the anemia state of pregnant women. Interestingly, Fe-content of iron pills varies among Primary Health Care facilities; however, again no difference in the anemia state due to different Fe-content, different suppliers, or the way pill is stored. Of note, ferro fumarate is easily oxidized to alkaline conditions or exposure to air, affecting the quality of ferrous fumarate [9]. When the drug is decomposed, the concentration of the active ingredient of the drug might change, and the drug dosage might be inaccurate, resulting in a decrease of effectiveness. This issue needs to be well monitored and evaluated by the government, as part of implementation of anemia control programs [18].

Compliance to iron supplementation might also be related to others age, education level, socioeconomic *c.q.* work status of the pregnant women [16]. Moreover, education is alleged to be a risk factor for anemia, especially the knowledge and the eating habits. Good knowledge on nutrition might increase the intake of iron supplement, resulting in less anemia prevalence in women with higher education [19]. Education greatly influences a person's ability to receive information; where the higher education is, the easier it is to receive information, especially about healthy living. Pregnancy in a young age or in older age might serve as a risk factor in relation in anemia incidence [15]. Ideally, pregnant women who work would have their own income and thus better financial status. High socioeconomic groups are usually more able to buy living necessities, especially nutrients needed during preparation for pregnancy and the pregnancy process than low socioeconomic groups. In developed countries, anemia prevalence is far lower (18%) than developing countries (41%) because of socioeconomic development, higher standard of living, better utilization of health facilities, and higher levels of education [18]. However, our study has revealed that there is no statistically significant difference in those factors with the anemia prevalence as confirmed in studies from other areas in Africa [20, 21] and in the neighboring country Malaysia [22]. For further increasing in the adherence of mothers taking iron supplement pills, the role of health workers involvement is indeed needed to help mothers making a self-reported compliance [23].

Moreover, other risk factors involved are the number of gravidity and parity [24]. Women with high parity have low or no iron storage because they have run out by repeated pregnancies so that women with multiparity are more likely to experience anemia. Our study has an incidence of anemia of 26.4% in the gravidity group 1–3, and there is no significant difference (*p* 0.957), in line with the study in Sudan showing no significant relationship between parity and the incidence of anemia in women of childbearing age [20]. However, the conflicting result has shown that parity is associated with the incidence of anemia; parity at risk especially >3 parities has a greater risk of anemia [22].

The condition of the mother and fetus in the womb is influenced by the nutritional status of the mother, both before pregnancy and during pregnancy. When nutritional status of the mother is not good, it may cause nutritional deficiencies that impact on the occurrence of complications during pregnancy that endanger the mother and fetus [25]. Our study has shown that the incidence of anemia in mothers tends to occur in women with BMI < 18.5 and normal BMI compared with BMI > 25. Pregnant women who have high BMI has a higher tendency for iron intake, and in lower BMI, malnutrition occurs and the amount of nutrients needed to maintain the body's organs and tissues is lacking to remain healthy and function properly [26].

Most of the midwife practices in Indonesia have used Hb Sahli method or finger prick test. Our previous study has shown that the finger prick test for Hb measurement has resulted in a higher anemia prevalence [13], which may lead to iron over therapy. Blood count examination has a better accuracy and the indices have more added values *i.e.* in detecting IDA or *β*-TT or carrier [27, 28]. Assessment of MCV and MCH as the first step of screening for the anemia type in pregnant women is, therefore, highly recommended [29], especially because Indonesia is located in the thalassemia belt area. Special attention has been raised in the study because there are no data available yet about thalassemia carriership from Kupang, eastern part of Indonesia. Interestingly, after iron supplementation in this study, there are high numbers of anemic pregnant women with low SLI. Evenmore, there are also high numbers of nonanemic pregnant women with low SLI. Taken together, in total, there are 37.2% suspected hemoglobinopathy carriers in the eastern part of Indonesia, compared to 5.7% suspected carriers in the western part as shown in our previous study [13]. Even though our study site is a malaria-endemic area, our respondents are not infected by malaria; thus, the cause of this infection has been eliminated. Further test to confirm possible anemia due to *β*-TT is required by HbA2 analysis and DNA examination.

Our study faces several limitations, among others that we conduct cross-sectional study because of incomplete pre-pregnancy data, such as Hb concentration and weight data at admission and during antenatal visits. Therefore, the result on iron supplementation is not well controlled; the follow-up study design should be thus performed.[30]. Furthermore, CBC is not examined as a regular Hb examination in PHCs, but in a finger prick test. The previous study has shown a very different result of anemia status between those two methods [13]. Because Kupang is a remote area where Hb electrophoresis examination and molecular DNA analysis are lacking, suspected *β*-TT cannot be confirmed; however, this may lead to another research area. Other limitation is that the self-designed questionnaire is lacking validity and reliability because of a dichotomous answer. An iron supplementation adherence scale might be of valuable information and need to be developed based on education and cultural belief.

## 5. Conclusion

Our study has shown that the prevalence of anemia among pregnant women in Kupang, eastern part of Indonesia, is still as high as 34.3% even after iron supplementation, and based on Shine and Lal index, 14.7% is because of suspected iron deficiency and 19.6% is because of suspected *β*-TT. In low-source area, Shine and Lal index might be of a great use to help midwives in predicting *β*-thalassemia carrier. Together with obstetricians, an integrative approach and counseling among pregnant women with *β*-thalassemia carrier and their partners will increase thalassemia awareness and birth management.

## Figures and Tables

**Table 1:** Demographic and clinical characteristic of pregnant women in three primary health care facilities in Kupang, Eastern Indonesia.

Clinical characteristic	Hb concentration	Total	*p* value	OR	(95% CI)
	<10.5 g/dL	≥10.5 g/dL			
Age; years old					
<20	2	3	5	0.544^*∗*^	n.d.
20–35	28	50	78		
>35	5	14	19		
Education					
Basic	5	8	13	0.061^*∗∗*^	n.d.
Middle	25	38	63		
High	5	21	26		
Occupation					
Housewife	30	49	79	0.149	n.d.
Working	5	18	23		
Gravidity					
1–3	27	52	79	0.957	n.d.
>3	8	15	23		
Trimester					
Second (14–26 week)	2	10	12	0.170	n.d.
Third (>26 week)	33	57	90		
BMI (kg/m^2^)					
<18.5	4	3	7	0.002^*∗∗∗*^	4.38
18.5–24.9	24	29	53		(1.68–11.39)
≥25	7	35	42		
Total	35	67	102		

*Note*. n.d., not determined. Anemia in pregnancy status was defined as Hb concentration <10.5 g/dL. Anemia status was not significantly associated (*p* > 0.05) with ^*∗*^ age in risk (<18 and >35 vs. 20–35 years old) or ^*∗∗*^ education (low and middle vs. high). Anemia status was significantly associated (*p* < 0.05) with ^*∗∗∗*^ body mass index in pregnancy (<25 vs. ≥25).

**Table 2:** Respondents' compliance with iron supplementation.

Questions	*n*	%
(1) How many iron pills have been given in your first visit?		
(a) >30 tablet	102	100
(b) <30 tablet	—	—
(2) How often do you take iron pills?		
(a) Daily	102	100
(b) Weekly	—	—
(3) When do you take the iron pills?		
(a) In the evening	95	93.1
(b) In the morning	7	6.9
(4) What do you drink together with the iron pills?		
(a) Mineral water	102	100
(b) Coffee/milk/tea	—	—
(5) Where do you store the iron pills at home?		
(a) In the storage place	101	99
(b) Somewhere in the desk	1	1
(6) Do you sometimes forget to take the iron pills?		
(a) Yes, sometimes	63	61.8
(b) Never	39	38.2
(7) What is the reason of not taking the iron pills?		
(a) Forgotten	66	64.7
(b) Others, e.g., nausea (*n =* 1), dizzy (*n =* 1), don't like it (*n =* 1), no reason (*n =* 33)	36	35.3
(8) How many iron pills left in this month?		
(a) ≥6 pills	76	74.5
(b) <6 pills	26	25.5
(9) How often you have difficulty of taking the iron pills?		
(a) Sometimes	23	22.5
(b) Never	79	77.5
(10) When you feel good, do you stop taking the iron pills?		
(a) Yes	37	36.3
(b) No	65	63.7

**Table 3:** Relationship between compliance, iron content, and Shine and Lal index with anemia among the study participants.

	Hb concentration		*p* value
	**<10.5 g/dL**	**>10.5 g/dL**	
*Questionnaire*			
Q6: Forgetfulness to take the iron pills			
Some time	25	38	0.147
Never	10	29	
Q8: Pill intake in %			
60–80%	23	53	0.141
>80%	12	14	
Q9: Difficulty in taking the pills			
Sometimes	10	13	0.293
Never	25	54	
Q10: Stop in taking the pills when feel good			
Yes	17	20	0.062
No	18	47	
*Fe content*			
∼75% (PHC 2and3)	34	61	0.247
100% (PHC1)	1	6	
*Shine and Lal index* ^*∗*^			
≥1530	15^a^	49	n.d.
<1530	20^b^	18^c^	
Total	35	67	

*Note*. Anemia in pregnancy was defined as Hb concentration <10.5 g/dL. n.d. not determined; PHC, primary health care. Statistically significance was set when *p* < 0.05. ^*∗*^Descriptively presented, ^a^Shine and Lal index ≥1530 in anemia indicated iron deficiency anemia (IDA); ^b^Shine and Lal index <1530 in anemia indicated *β*-thalassemia carrier (*β*-TT); ^c^Shine and Lal index < 1530 in nonanemic individual required further examination to exclude *β*-thalassemia carrier or other hemoglobinopathies.

**Table 4:** The type of anemia distribution among pregnant women in second and third trimester stratified based on body mass index and anemia severity.

	Anemia		No anemia		Total (*n*102)
	SLI ≥ 1530	SLI < 1530	SLI < 1530	SLI ≥ 1530	
	IDA (*n*15)	Susp. *β*-TT (*n*20)	Susp. *β*-TT (*n*18)	Normal (*n*49)	
2^nd^ trimester (*n* = 12)					
BMI < 18.5	—	1	1	—	2
BMI 18.5 to <25	—	1	2	3	6
BMI ≥ 25	—	—	—	4	4
3^rd^ trimester (*n* = 90)					
BMI < 18.5	1	2	—	2	5
BMI 18.5 to <25	12	11	5	19	47
BMI ≥ 25	2	5	10	21	38
2^nd^ trimester (*n* = 12)					
No anemia	—	—	3	7	10
Mild anemia	—	2	—	—	2
3^rd^ trimester (*n* = 90)					
No anemia	—	—	15	42	57
Mild anemia	15	14	—	—	29
Moderate anemia	—	4	—	—	4

*Note*. BMI, body mass index; IDA, iron deficiency anemia; susp. *β*-TT, suspect *β*-thalassemia trait; SLI, Shine and Lal index.

## Data Availability

Data used to support the study are available from the corresponding author upon request.
